# Development and Validation of an Immune-Related Long Non-coding RNA Prognostic Model in Glioma

**DOI:** 10.7150/jca.53831

**Published:** 2021-05-19

**Authors:** Xiaowei Qiu, Yehong Tian, Jingnan Xu, Xin Jiang, Zeyu Liu, Xuewei Qi, Xin Chang, Jianxin Zhao, Jinchang Huang

**Affiliations:** 1 Beijing University of Chinese Medicine Third Affiliated Hospital, Beijing, China.; 2 Institute of Acupuncture and Moxibustion, Shaanxi University of Chinese Medicine, Shaanxi, China.

**Keywords:** glioma, immune, LncRNA, prognosis

## Abstract

**Background:** Long non-coding RNAs (lncRNAs) play an important role in the immune processes of glioma. Immune related lncRNAs (IRlncRs) may be a critical prognosis in patients with glioma. The current study aimed to construct a glioma immune-related prognosis model by IRlncRs.

**Methods:** Transcriptome RNA-sequencing data of glioma were obtained from The Cancer Genome Atlas (TCGA) and an immune‑related risk score (IRRS) model was constructed by Lasso and multivariate Cox regression analysis. Receiver Operating Characteristic (ROC) curves were used to assess the sensitivity and specificity of the prognosis on IRRS. A predictive nomogram and a time-dependent ROC curve was performed in training and validation cohort. We explored the relationships between survival‑related IRlncRs (sIRlncRs) and clinicopathologic parameters. Functional annotation of the sIRlncRs was investigated by gene set enrichment analysis (GSEA) and principal component analysis (PCA). The relationships between IRRS model and immune cell infiltration and co-expression network analysis among the sIRlncRs were performed for molecular mechanism study.

**Results:** A total of 10 sIRlncRs were enrolled to build IRRS model. The IRRS was identified as an independent prognostic factor and correlated with the overall survival (AUC =0.880). The nomogram was constructed successfully with IRRS, age and grade as variables. Immune cell infiltration analysis indicated that B cells, neutrophil, dendritic and macrophage cells were positively correlated with IRRS. PCA and GSEA illustrated that the lncRNA signature enrolled the IRRS model was closely related to immune status. Additionally, co-expression network showed that there was a strong correlation between 10 sIRlncRs at the transcriptional level.

**Conclusion:** We successfully constructed a remarkable clinical model of sIRlncRs with potential prognostic value for glioma patients, which provides an insight into immunological research and treatment strategies of glioma.

## Introduction

Glioma is the most common primary cancer of the central nervous system caused by astrocytes or astrocyte precursor cells, which accounts for approximately 81% of all malignant intracranial tumors 1. Every year, about 100,000 people worldwide are diagnosed as gliomas 2. Based on the degree of cell malignancy, gliomas are classified as grade 4 and the degree of malignancy increases with the enhancement of grade 3. The low-grade glioma (LGG) is only account for 15% of all gliomas 3, 4 and the 5-year survival rate of patients with LGG is 30-70%. However, glioblastoma (GBM), the most lethal glioma, accounts for 70% of all glioma, the median survival time of patients with GBM is only 9-12 months and the 5-year survival rate is 0.05% to 4.7% 5, 6, which indicated that the prognosis of most glioma patients is still poor. Therefore, it is necessary to further study the clinical diagnosis and treatment methods of glioma, explore new risk factors and molecular markers, and develop new therapeutic targets, which have improved the clinical prognosis of patients with glioma.

Long non-coding RNAs (lncRNAs) were once considered as RNA transcripts with a base length greater than 200 nucleotides and lacking protein-coding ability. Recent studies have shown that lncRNAs play key roles in a wide range of cellular biological processes by regulating gene expression at the transcriptional, post-transcriptional and epigenetic levels 7, 8. Moreover, lncRNAs significantly affect the immune processes of tumors, including antigen exposure and recognition, as well as immune infiltration 9. Notably, multiple aberrant expressions of lncRNAs play indispensable roles in the onset and progression of glioma malignancy, including the stemness, proliferation, angiogenesis and drug resistance 10, 11, which have important clinical implications in the glioma diagnosis of sub-classification 12 and prognostication 12, 13. For example, the overexpression of lncRNA PXN-AS1-L is associated with unfavorable prognosis in patients suffering from glioma 14. One of the distinctive features of lncRNAs is their highly tissue and cell type specific expression patterns 15, which could accurately classify different subtypes of glioma and predict responses to treatments. In addition, lncRNAs transmitted from cell to cell participate in intercellular communications for maintaining microenvironment homeostasis or mediating tumor metastasis 16.

Currently, cancer immunotherapy is a validated and critically important approach for treating patients with cancer, particularly immune checkpoint inhibitors including programmed death 1, PD-1 ligand, and cytotoxic T lymphocyte-associated antigen 17. Although it is also used in glioma, the effect is not satisfactory 18. Immune-related genes (IRGs), such as immune related lncRNAs (IRlncRs), are abundant in immune cells and tumor immune microenvironment, which are involved in the tumorigenesis and tumor progression of glioma 19. Therefore, the study of the immune-related molecular mechanism in glioma is of great significance for the treatment and prognosis of patients with glioma.

In this study, we designed to investigate the clinical role of IRlncRs in prognostic evaluation of glioma. We extracted a series of IRlncRs in tumor immune microenvironment as predictive factors and further constructed a prognosis model to evaluate the relationship between the model with overall survival (OS) in combination with their clinicopathological features. The study would be helpful to establish a personalized quantitative predictive model and elucidate the potential mechanism of IRlncRs in glioma.

## Methods

### Publicly available mRNA data

A series of transcriptome RNA-sequencing data of human glioma samples were downloaded from the TCGA data portal (https://porta l.gdc.cance r.gov/), which were updated to November 11, 2019. The inclusion and exclusion criteria of data for the further analysis were as follows. The inclusion criteria: 1. Data of gliomas including Low grade (LGG) gliomas and glioblastomas (GBM); 2. The gene expression of fragments per kilobase million (FPKM) were normalized in transcriptome. The exclusion criteria: 1. Survival time is less than or equal to 30 days; 2. Clinical data is null or information is not clear; 3. Normal or paracancerous tissue samples; 4. Annotation information is not matched with the samples information. 5. Duplicate data. The data was prepared to further analyze after processing. RNA-seq results were combined into a matrix file using a merge script in the Perl language (http://www.perl.org/). Next, the script in the Perl language was also used to convert the Ensembl ID of genes into a matrix of gene symbols. Because the data were downloaded directly from public databases and we strictly followed the publishing guidelines provided by TCGA, no ethical approval was required.

### IRlncRs acquisition

The Molecular Signatures Database V4.03 (Immune system process M13664, Immune response M19817, http://www.broad institute.org/gsea/msigdb/index.jsp) was used to specify IRGs participation in the immune process. IRGs were used to establish the immune score of glioma gene by Gene Set Enrichment Analysis (GSEA). Pearson correlation analysis was used to evaluate the association by co-expression analysis between immune gene and lncRNA in glioma patients (“limma” R package with a standard of |r| > 0.7 and *p* < 0.001).

### Construction and validation of the immune-related risk score (IRRS) model

Survival‑related IRlncRs (sIRlncRs), associated with clinical outcomes in glioma patients, were screened by a univariate Cox proportional regression model using “survival” R package (*p* < 0.001). Next, the least absolute shrinkage and selection operator (LASSO) method was used for the selection of variables by a Cox regression model to determine significant prognostic lncRNAs with “glmnet” R package. To make our model more optimized and practical, a stepwise Cox proportional hazards regression model was used carried out by “survival” and “glmnet” R packages. Finally, a risk score formula was calculated by considering the expression of optimized genes and correlation estimated Cox regression coefficients:


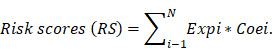


All glioma patients with survival time were randomly divided into the training and validation cohort at a ratio of 7:3 using the “caret” R package. The median RS served as a cutoff value to classify the patients into the high- or low-risk groups in each cohort. ROC curves were utilized to assess the sensitivity and specificity of the prognosis by “time ROC” R package. Kaplan-Meier plot was applied to visualize the OS probability of glioma patients between high-risk group and low-risk group in which survival differences were evaluated by a two-sided log-rank test.

### Identification of independent prognostic factors

We performed rough analysis to identify potential risk factors in univariate Cox regression analysis. After selecting the potential risk factors, we used three selection procedures (forward, backward, and stepwise) for multivariate Cox regression analysis to select the most suitable model. The statistical significance level was 0.05 used to select the variables. Next, the “survival ROC” R package was used to draw the ROC curves of multiple items (containing age, gender, grade and IRRS) to evaluate the accuracy of the predicted survival probability.

### Construction and validation of a predictive nomogram

The nomogram was performed by “rms” R package consisting of independent prognostic factors and related clinical parameters as variables based on multivariate Cox regression analysis in training and validation cohort. According to the different variables feature, a horizontal straight line was draw to ascertain the points for each variable, and the total points of each patient were calculated by adding the points of all variables together, which were normalized to a distribution from 0 to 100. The estimated OS rates at 1, 3, and 5 years of glioma patients were calculated by drawing a vertical line between the total point coordinate axis and each prognostic coordinate axis. The concordance index (C-index) by “survcomp” R package and calibration plot by “rms” R package were applied to validate the performance of the nomogram in training and validation cohort (by a bootstrap method with 1,000 resamples).

### Principal component analysis (PCA) and Gene set enrichment analysis (GSEA)

PCA was carried out with “scatterplot3D” R package to demonstrate the expression of glioma samples in low-risk and high-risk groups. GSEA was used to detect the different functional phenotype between the low-risk group and high‐risk group in the TCGA cohort.* p* < 0.05 was considered statistically significant.

### Correlation analysis between immune‑related lncRNA signature and immune cells infiltration

To explore the associations between immune related lncRNA signature and immune cells infiltration, we employed Tumor Immune Estimation Resource (TIMER) 20 (https://cistrome.shinyapps.io/timer/), a useful resource for comprehensive analysis of tumor infiltrating immune cells. TIMER algorithm allows users to estimate the composition of six tumor infiltrating immune cells subsets (B cells, CD4+ T cells, CD8+ T cells, macrophages, neutrophils, and dendritic cells). The immune infiltrate levels of glioma patients were derived from TIMER website. The correlation between the prognostic model and six tumor-infiltrating immune cells were conducted in R.

### IRlncRs co‑expression analysis

To define the strength of these transcriptional level relationships, co-expression relationships among these IRlncRs were calculated based on RNAseq levels, and Pearson correlations between genes were calculated by “corrplot” R package.

### Statistical analysis

All data analysis was carried out by R version 4.0.2 and corresponding packages. For all data, statistical significance was indicated in the figures as follows: *p < 0.05, **p < 0.01, ***p < 0.001.

## Results

### Acquisition of glioma expression and clinical data

Fragments per kilobase per million normalized expression used as the RNA-seq results of 698 glioma samples and 5 normal samples were obtained from TCGA. In addition, there were 629 glioma patients with survival information enrolled from TCGA dataset. The transcriptome data was processed to convert the data ensembl ID into gene names and divided into lncRNA and mRNA. Additionally, we identified 331 glioma IRGs from the Immune system process M13664 and Immune response M19817 of Molecular Signatures Database.

### The correlation analysis of IRlncRs and prognosis

**Figure 1:**
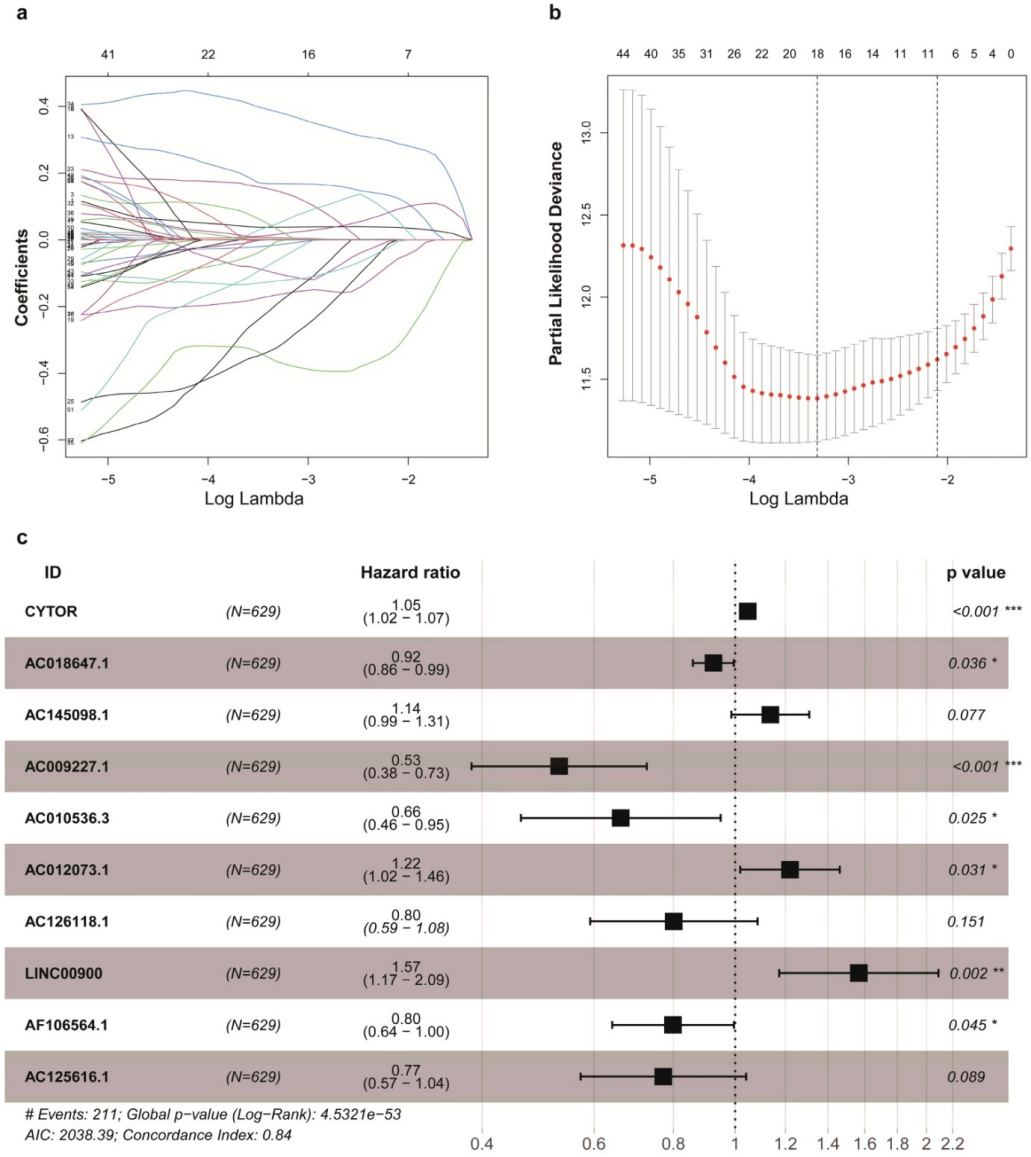
Identification of IRlncRs associated with glioma prognosis. a, b. Eighteen IRlncRs were identified by the lasso regression analysis. c. Ten sIRlncRs were identified for the construction of a prognostic model by the multivariate Cox regression analysis.

Firstly, a total of 92 lncRNAs were identified to be the IRlncRs by correlation analysis of co-expression (Supplementary Table 1). Then, we identified 63 IRlncRs (Supplementary Table 2) by the univariate Cox proportional regression model. Next, 18 IRlncRs were identified by lasso analysis (Figure 1a, 1b). Lastly, 10 sIRlncRs including CYTOR, AC145098.1, AC012073.1, LINC00900, AC009227.1, AC010536.3, AC125616.1, AF106564.1, AC126118.1 and AC018647.1, were identified for the construction of the prognostic model by multivariate Cox regression analysis to establish the IRRS model (Figure 1c). The regression coefficients of CYTOR, AC145098.1, AC012073.1 and LINC00900 were positive, while the regression coefficients of AC009227.1, AC010536.3, AC125616.1, AF106564.1, AC126118.1 and AC018647.1 were negative.

### Construction and validation of the IRlncRs signature for survival prediction

**Figure 2:**
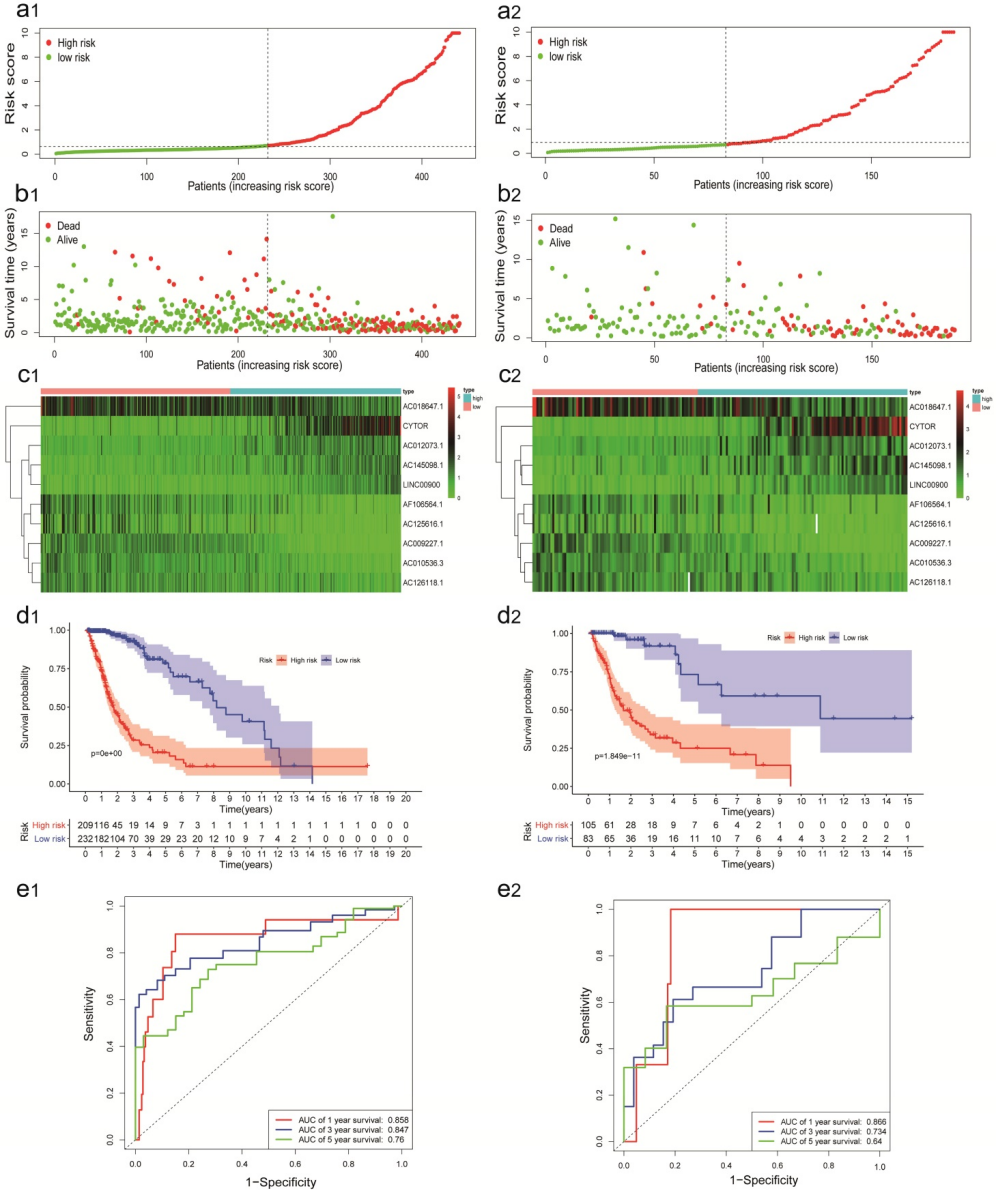
Construction and validation of the IRRS model for survival prediction in training cohort (a1, b1, c1, d1, e1) and validation cohort (a2, b2, c2, d2, e2). a1, a2. The risk score distribution of glioma patients. b1, b2. Survival status and duration of patients. c1, c2. Heatmap of the ten immune‐related genes expression in glioma patients. d1, d2. The OS in the high-risk group was significantly worse than that in the low-risk group. e1, e2. Receiver operating characteristic curve (ROC) analysis predicted OS probability based on the IRRS.

A total of 629 glioma patients with survival information were randomly assigned to training cohort (n=441) and validation cohort (n=188). The IRRS of each glioma patients in the TCGA were calculated, and these patients were divided into the high-risk group and the low-risk group by median IRRS (Figure 2a1). The mortality rate constantly increased with the higher IRRS (Figure 2b1). With the enhancement of IRRS, the expression levels of CYTOR, AC012073, AC145098.1.1 and LINC00900 were individually elevated, while the AC009227.1, AF106564.1, AC125616.1, AC018647.1, AC010536.3, and AC126118.1 expressed decreasingly (Figure 2c1). Also, the OS probability of the high-risk group was significantly shorter than that of the low-risk group based on Kaplan-Meier analysis and log-rank test (*p* < 0.001, Figure 2d1). The AUC values of time-dependent ROC curves on 1-, 3- and 5- years were 0.858, 0.847 and 0.760 in training cohort, which indicated higher sensitivity and specificity of the IRRS model to predict OS of patients with glioma (Figure 2e1).

The predictive power of the IRRS model was confirmed by validation cohort. The IRRS, mortality rate, the expression levels of 10 sIRlncRs and OS analysis in validation cohort with the same trend in training cohort (Figure 2: a2, b2, c2, d2). The AUC values of time-dependent ROC curves on 1-, 3- and 5- years were 0.866, 0.734 and 0.640 in validation cohort (Figure 2e2).

### Independent prognostic factor identification and prognostic nomogram construction

**Figure 3:**
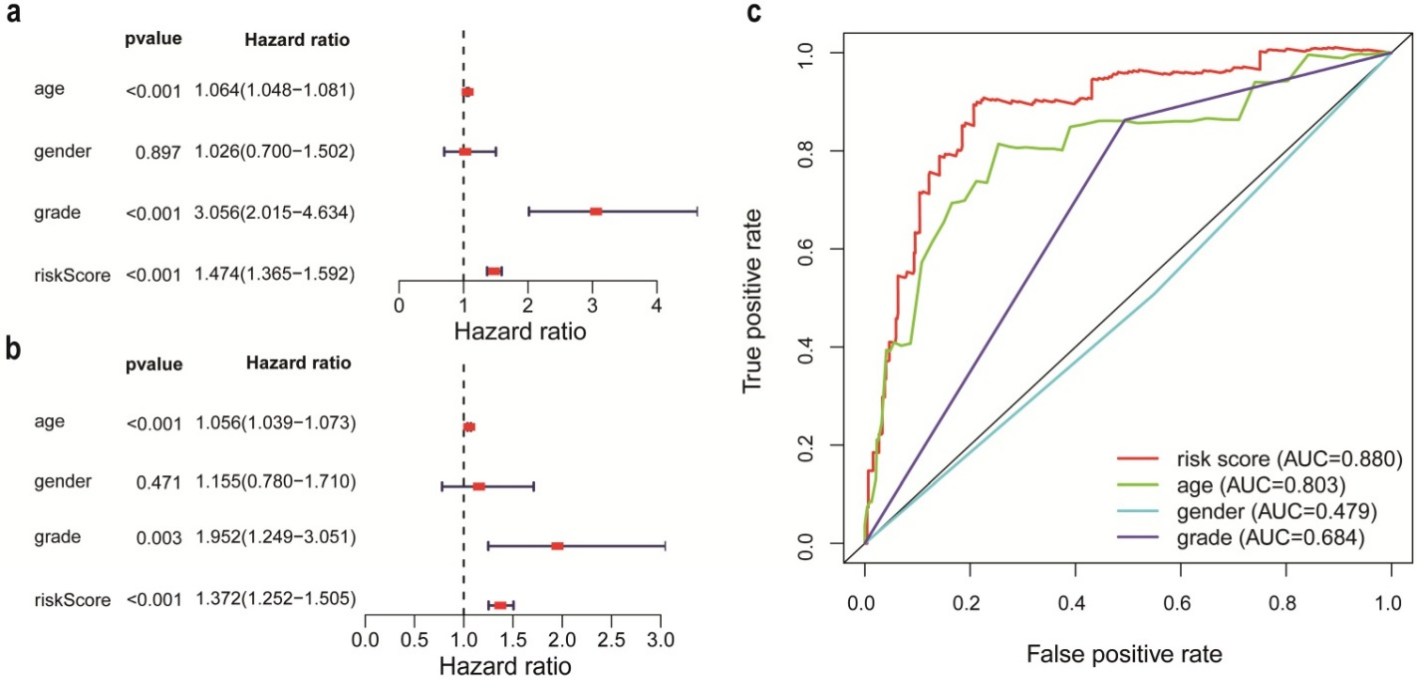
The analysis of independent prognostic factors in patients with glioma. a. Univariate Cox regression analysis. b. Multivariate Cox regression analysis. c. Receiver operating characteristic (ROC) curve of risk score and clinicopathologic features.

**Figure 4:**
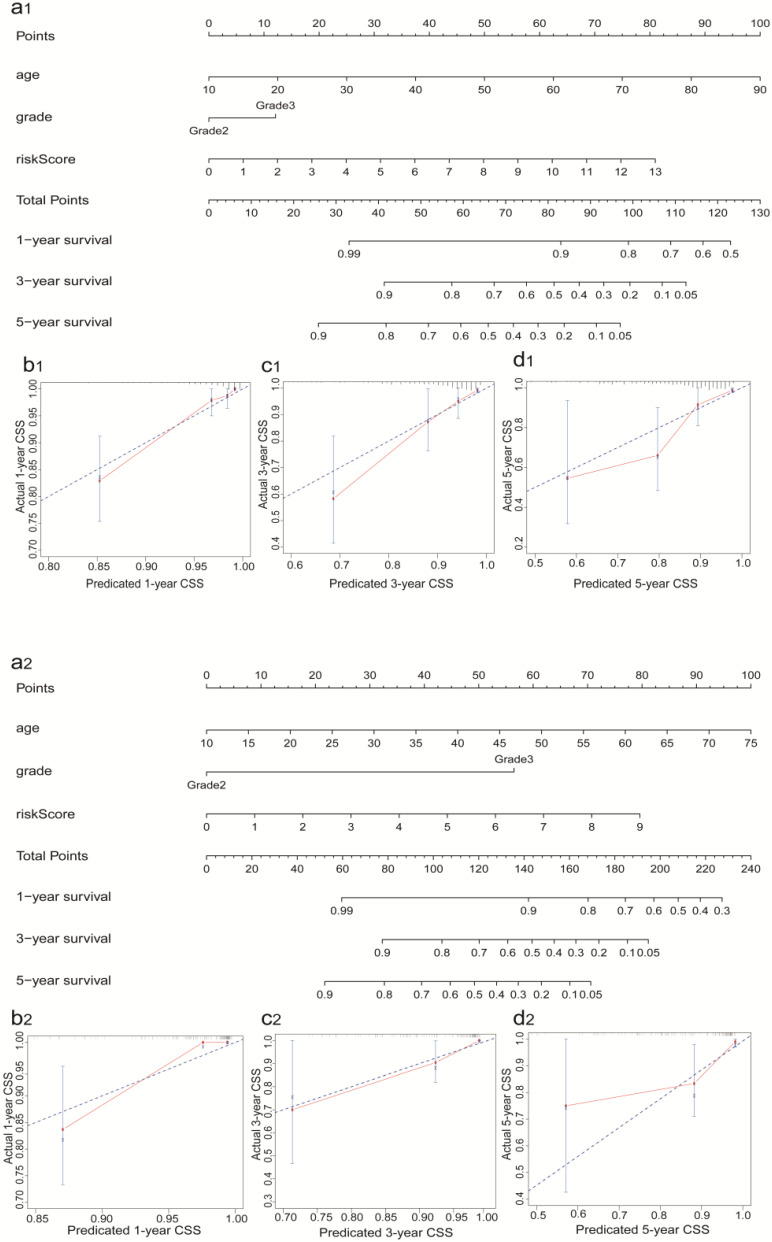
Construction and validation of the Prognostic Nomogram in training (a1, b1, c1, d1) and validation (a2, b2, c2, d2) cohort. a1, a2. Construction of the nomogram was based on the IRRS, age and grade. b1 and b2, c1 and c2, d1 and d2. Calibration plot for the internal validation of the nomogram.

According to the criteria of inclusion and exclusion, there were 476 glioma samples with detailed clinical information enrolled. The baseline characteristics of these patients were shown in Table 1. We identified the independent prognostic factors in patients with glioma by univariate and multivariate analysis in entire cohort. The results indicated that age, grade and IRRS can be independent predicated factors affecting prognosis (Figure 3a, 3b). The AUC values of IRRS, age and grade were 0.880, 0.803 and 0.684 respectively (Figure 3c). To provide the clinician with a quantitative method for predicting the probability of 1-,3- and 5-year OS in glioma, a nomogram incorporating IRRS, age and grade as variables was constructed in training cohort (Figure 4a1) and validation cohort (Figure 4a2). The calibration plots, the predictive curves were close to the ideal curves, showed that the performance of the nomogram was the best in predicting the 1-, 3-, and 5-year OS in training cohort (Figure 4: b1, c1, d1) and validation cohort (Figure 4: b2, c2, d2). The C-index of our nomogram reached 0.843 (95% CI: 0.801-0.884) in training cohort and 0.852 (95% CI: 0.795-0.909) in validation cohort, respectively. The nomogram can be used to predict the OS rate of different patients according to their own conditions to improve the prediction efficiency and accuracy.

### Correlation between sIRlncRs and clinicopathologic indicators

**Figure 5:**
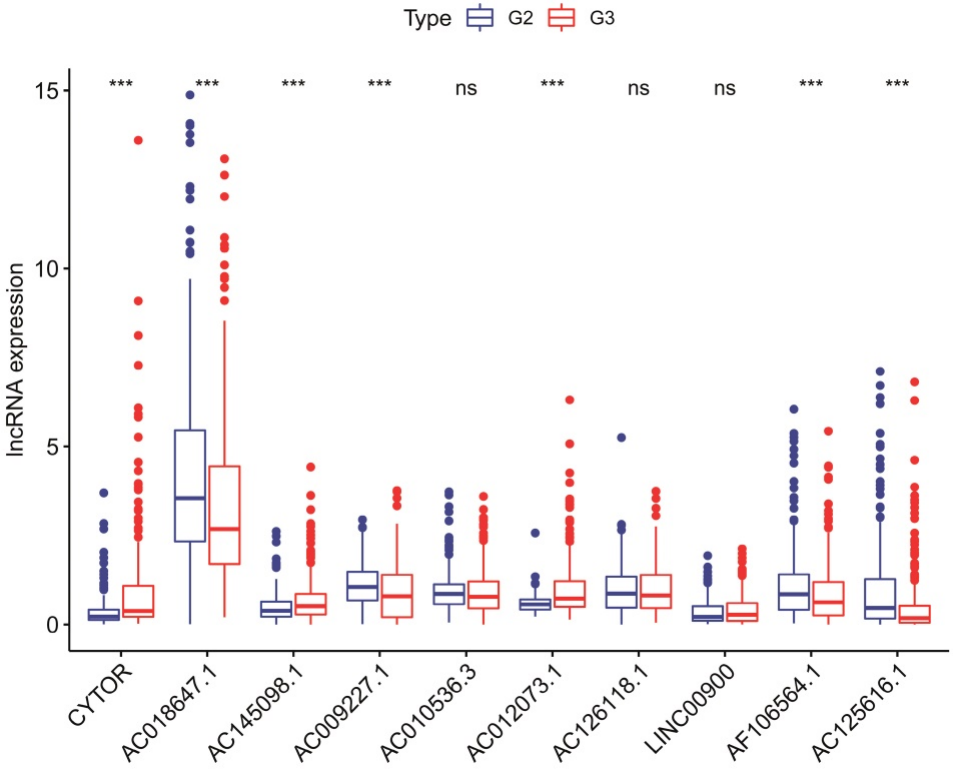
The relationships between the 10 sIRlncRs and clinical grade in patients with glioma in entire cohort. ns, no significance.

To investigate the relevance of the sIRlncRs and clinicopathological features of glioma, we analyzed the correlation between IRRS and clinical grade in entire cohort. The clinical characteristics of patients with high-risk score and low-risk score were shown in Table 1. Ultimately, we found the expression of CYTOR, AC145098.1 and AC012073.1 enhanced in the grade 3 (G3) than grade 2 (G2); the expression levels of AC018647.1, AC018647.1, AF106564.1 and AC125616.1 were decreased in the grade 3; while there were no differences in the expression of AC010536.3, AC126118.1, and LINC00900 between grade 3 and grade 2 (Figure 5).

### The immune status of the low and high‑risk group

**Figure 6:**
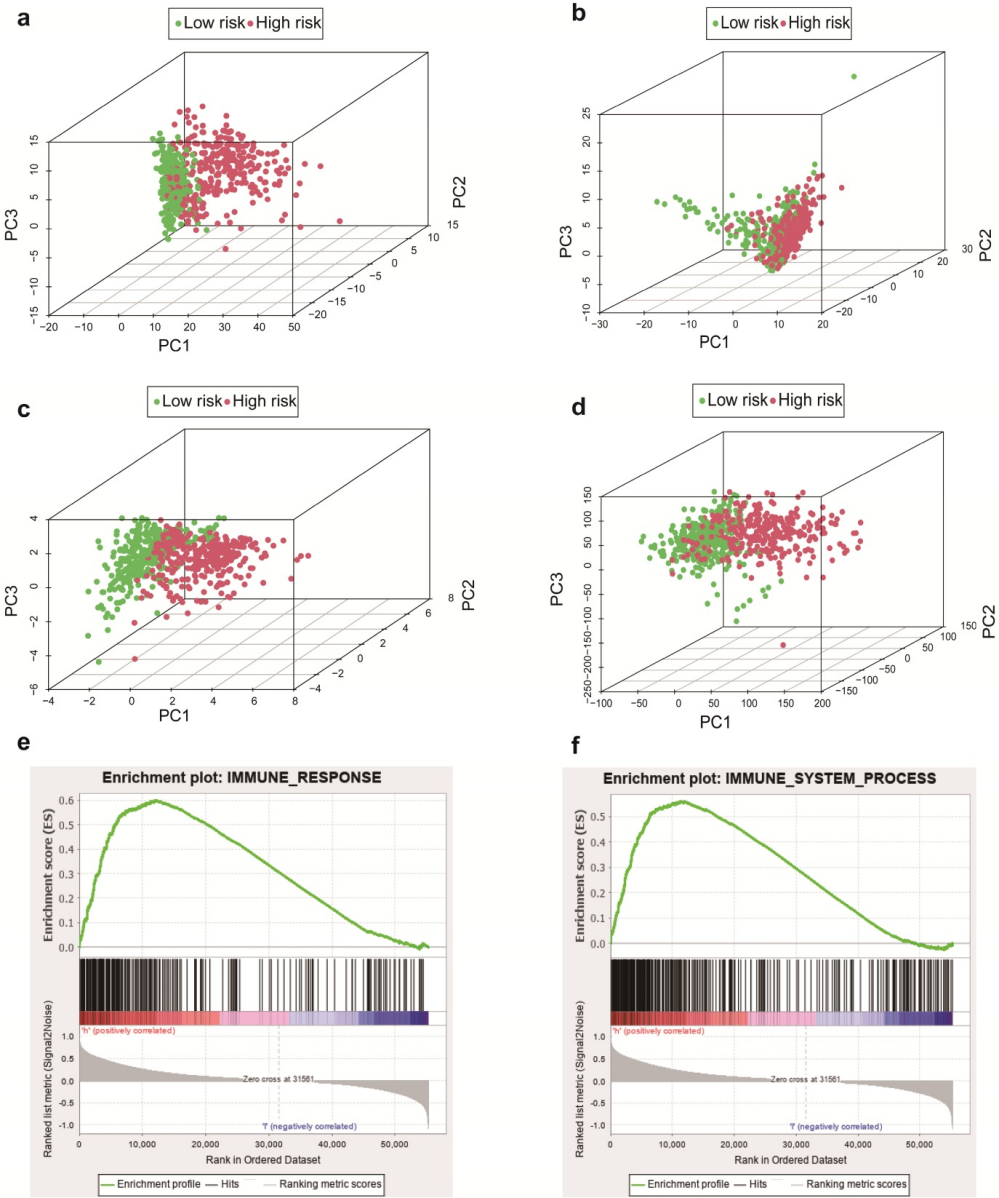
Principal components analysis (PCA) and Gene set enrichment analysis (GSEA) in entire cohort. The high‐risk group and low‐risk group demonstrated different immune status. PCA between high‐risk group and low‐risk group based on the sIRlncRs gene sets (a), IRlncRs gene sets (b), IRGs sets (c), and all gene sets (d), respectively. The red and green dots represent high-risk and low-risk gene, respectively. GSEA indicated significant enrichment of immune‐related phenotype in the high‐risk group (e and f).

**Figure 7:**
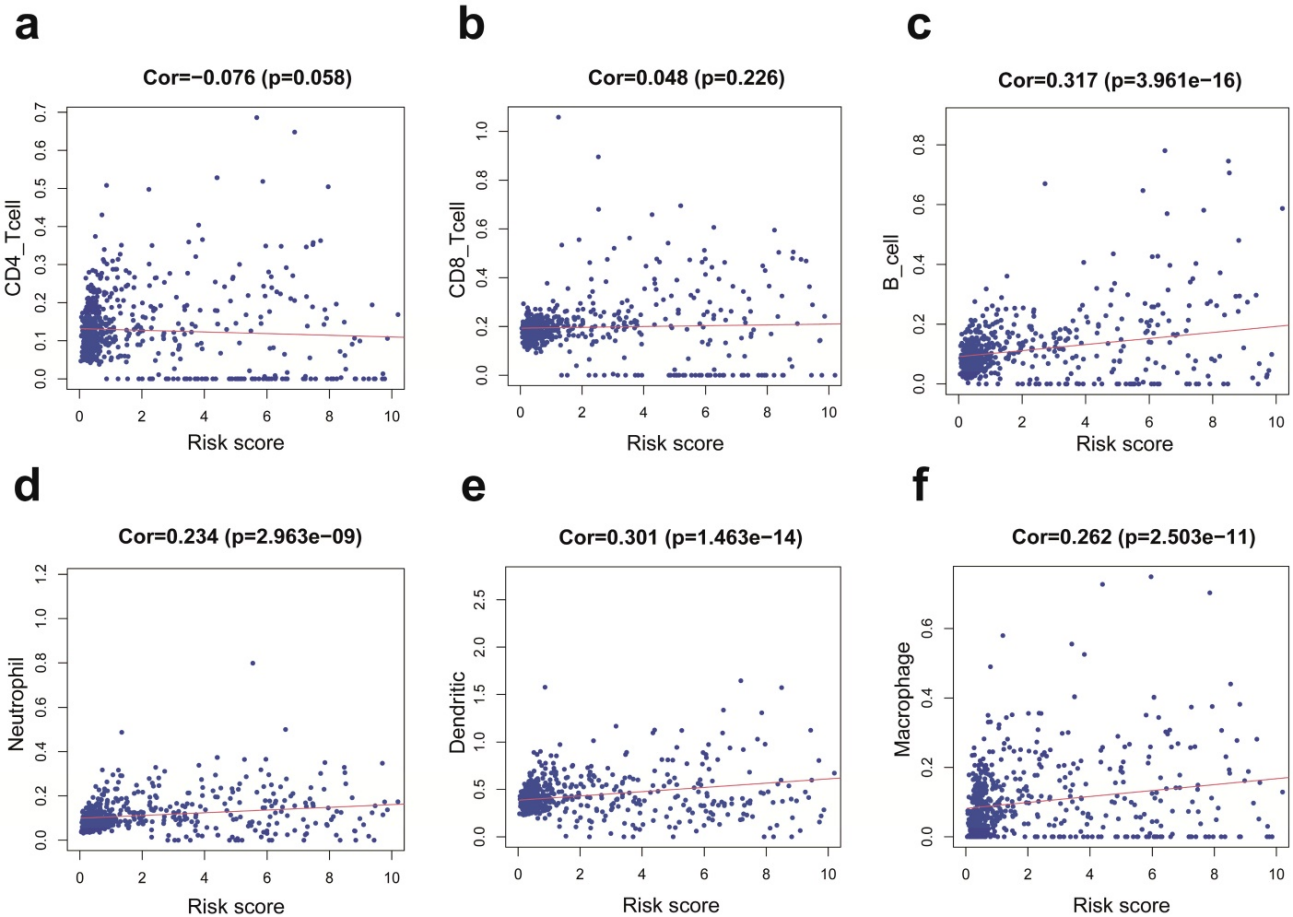
Relationships between the IRRS model and infiltration abundances of six types of immune cells in entire cohort. These immune cells include CD4-T cell (a), CD8-T cell (b), B cell (c), neutrophil (d), dendritic cell (e), and macrophage (f).

We employed the PCA to detect the different distribution patterns between the low-risk group and the high-risk group in entire cohort. In the sIRlncRs gene set (Figure 6a), the low-risk group and the high-risk group were separated into two parts, of which the low-risk group having the lower immune scores than the high-risk group. While we didn't detect the significant separation of the risk score based on the IRlncRs gene set (Figure 6b), IRGs set (Figure 6c) and all gene sets (Figure 6d). The results of GSEA further confirm that immune-related responses and processes are more common in high-risk groups (Figure 6e, 6f). The relationships between the risk score model and immune cell infiltration were investigated. As shown in Figure 7, B cells, neutrophil, dendritic cells and macrophage were positive correlated with risk score, respectively. However, no significant correlations were observed between CD4+ T cells, CD8+ T cells and risk score.

### Co‑expression of sIRlncRs

**Figure 8:**
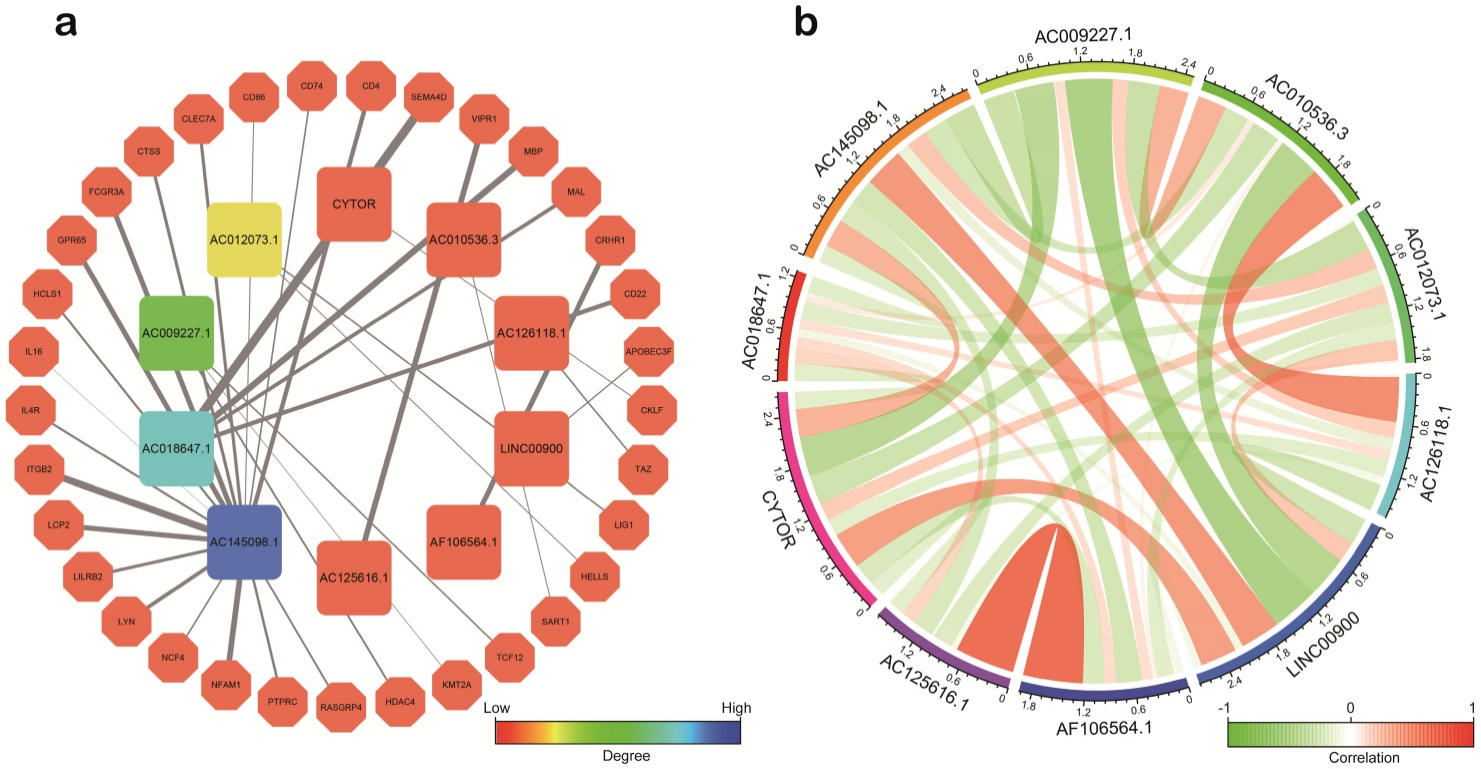
Correlation analysis of the ten sIRlncRs. a. Correlation of co-expression between sIRlncRs and immune gene at transcriptional level. The polygons represent immune gene, the squares represent sIRlncRs. The edges represent the relationship between sIRlncRs and immune gene. The width is proportional to the degree of correlation. b. Correlation analysis among 10 sIRlncRs. The red bands represent positive correlation, and the green bands show negative correlation. The width of the bands represents the degree of correlation between the sIRlncRs.

Based on co-expression analysis between lncRNAs and immune gene, we found AC145098.1 and AC018647.1 among the 10 sIRlncRs were closely associated with immune gene (Figure 8a). Meanwhile, as is shown in the Figure 8b, the 10 sIRlncRs had a close correlation between each other and formed a complex network of biological regulation, in which the highest positive correlation is AF106564.1 and AC125616.1 (0.72), while the highest negative correlation is LINC00900 and AC009227.1 (-0.54).

## Discussion

Glioma is the most invasive tumor of the human nervous system. Despite some major advances in treatment, including surgery, chemotherapy and radiation therapy, have been developed and widely used. Due to the aggressive growth of glioma, complexity of intracranial anatomy of the central nervous system and the presence of the blood-brain barrier 21,22, the treatment outcome of glioma patients remains unsatisfactory. Multiplied studies have revealed that the individual variation at the genetic level should be the cause of this phenomenon 23. As glioma is a heterogeneous disease characterized by variant morphologic and molecular altered, the prognosis and tumor response to therapy varied greatly between different individual patients with similar clinical risk factors 24.

During the past decades, lncRNA have attracted more and more attention from researchers, especially IRlncRs. LncRNAs exist in either the nucleus or cytoplasm, and play distinct functions depend on their subcellular localizations. In the nucleus, lncRNAs may participate in transcriptional regulation of gene expression and mRNA splicing. While in the cytoplasm, they could affect mRNA stability and regulate protein function 25. Moreover, lncRNAs exert their functions through multiple molecular mechanisms, such as binding with DNA to modulate gene transcription, acting as the ceRNA or miRNA sponges to regulate gene expression at posttranscriptional level, associating with proteins, and encoding functional small peptides 26. It has been confirmed that LncRNAs can function as molecular signaling mediators to regulate glioma phenotypes through mediating the expression of genes in NEAT1-WNT/β catenin 27 and CRNDE- mTOR signaling pathway 28. For instance, LncRNA HULC regulates ESM-1 through PI3K/Akt/mTOR signaling pathway and plays a critical role in pro-angiogenesis in human glioma 29. LINC01198 was proved to be up-regulated in glioma cell lines, and silenced LINC01198 inhibited glioma cell proliferation and accelerated cell apoptosis 30. Therefore, it is of great clinical significance to explore the potential therapeutic targets and accurate prognostic indicators of glioma.

In the current study, the parameters of screening lncRNAs to construct the prognosis model were apparently different from previous studies 31-33. In total, 10 sIRlncRs signature were included in the IRRS model to predict clinical prognosis. Furthermore, we analyzed the reliability and stability of the model by training and validation cohort, and results indicated the model was robust. Most glioma occurs with a 30-50% higher incidence in males than females and increase with age in adult 34. Risk of death enhanced 1.23-fold in recurrent glioma patients with decade increase in age, and 2.01-fold in initial histology of GBM 35. Generally, there is a poor prognosis in patients with high-grade glioma according to morphological criteria 36. Moreover, the immune related lncRNA signature is significantly related to survival of glioma 32. In our prognosis model, both univariate and multivariate Cox regression analyses were performed to identify independent prognostic factors of glioma patients among age, gender, grade and IRRS. Only age, grade and IRRS were selected as variables in the construction of nomogram in training and validation cohort to provide the clinician with a quantitative method for predicting the probability of 1-,3- and 5-year OS. The gender was excluded because its p value was higher than 0.05 in univariate and multivariate Cox regression analyses. (Figure 4: b1 and b2, c1 and c2, d1 and d2). Of note, the previous studies showed that some lncRNAs upregulated in glioma can promote the proliferation and invasion of glioma cells 37,38, while some lncRNAs downregulated in glioma may play a role in inhibiting tumor cell proliferation and promoting apoptosis 39. We also analyzed the relationship between sIRlncRs expression and clinical pathological grade, and further clarified the guiding role of prognosis model for clinical.

In the meantime, we additionally carried out the PCA, GSEA, the correlation between immune related signature and immune cell infiltration and the co expression gene network analysis, which were definite different from Zhou M 31 and pan YB's 33 study. We performed PCA to study the distribution patterns of all genes, IRGs, IRlncRs and sIRlncRs in low-risk and high-risk groups. According to the sIRlncRs set, the low-risk group and the high-risk group tend to be divided into two parts. The immune score of the low-risk group is lower than that of the high-risk group (Figure 6a). When PCA was performed according to all genes, IRGs, IRlncRs genome expression, there was no significant separation of immune status in these groups (Figure 6b, 6c, 6d). GSEA was applied to further verify the functional annotation. We found that there were more immune related reactions and processes in high-risk groups, with higher scores indicating the poor prognosis. PCA and GSEA analysis suggested that immune related biological reactions were more common in high-risk population of glioma. Previous studies have shown that immune microenvironment is closely associated with tumor prognosis. Multiple researchers have revealed the impact of the immune microenvironment on tumor cells 40,41. In our study, the results suggest that IRRS was positively correlated with the contents of B cells, neutrophils, dendritic and macrophage cells in the tumor immune microenvironment, which further indicated that the IRRS model constructed in this study has positive significance for clinical prognosis.

In addition, we also explored the potential molecular mechanism of the sIRlncRs expression in glioma. By co-expression network analysis, we found AC145098.1 and AC018647.1 in the ten sIRlncRs were closely associated with immune gene. Relationships among these sIRlncRs calculated based on RNAseq levels, we found that there was a strong correlation between 10 sIRlncRs at the transcriptional level. These results suggest that the risk assessment score based on the 10 sIRlncRs can contribute to identify high-risk patients from the same clinical or molecular characteristics to achieve individualized and appropriate treatment strategies.

In brief, we have achieved a group of different IRlncRs to construct and validate the prognosis model successfully. The significance of our study is helpful for validating previous results in which IRlncRs are of important clinical implications in patients with glioma. Despite our results provided reliable evidence for further analysis to study the occurrence and development of glioma, limitations inevitably affect our research. More datasets of whole genome sequencing in glioma disease are needed to validate prognostic model and potential biomarkers. The efficiency of these sIRlncRs signature in glioma remains to be further verified by functional and mechanism experiments *in vitro* and *in vivo*.

## Conclusion

In conclusion, we successfully constructed a sIRlncRs prognostic model with powerful predictive function and analyzed the potential mechanism of the 10-sIRlncRs included in the model. The study has certain guiding value for the analysis of the pathogenesis and clinical treatment of glioma, and these sIRlncRs may become new biomarkers and therapeutic targets for inhibiting the development of glioma.

## Supplementary Material

Supplementary tables.

## Figures and Tables

**Table 1:** Baseline characteristics of glioma patients in TCGA

Clinical Characteristics	All	High risk	Low risk	*p*-value
No.	476	161	315	-
Age, median (IQR)	41 (33 to 53)	47 (35 to 59)	39 (32 to 49)	<0.001
Gender, No. (%)				
Female	216 (45%)	69 (42.86%)	147 (46.67%)	0.43
Male	260 (55%)	92 (57.14%)	168 (53.33%)	
Grade, No. (%)				
G2	231 (49%)	47 (29.19%)	184 (58.41%)	<0.001
G3	245 (51%)	114 (70.81%)	131 (41.59%)	
Futime, median (IQR)	590 (375 to 1105.5)	546(326 to 915)	622 (407 to 1164)	0.0363
Fustat, No. (%)				
Live	369 (78%)	96 (59.63%)	273 (86.67%)	<0.001
Death	107 (22%)	65 (40.37%)	42 (13.33%)	

Note: IQR, interquartile range; Futime, survival time; Fustat, survival state. p-value: p value represents the difference of clinical characteristics between high and low risk groups.

## Abbreviations

AUC: area under the curve
GBM: glioblastoma
GSEA: gene set enrichment analysis
IRGs: immune-related genes
IRlncRs: immune related lncRNAs
IRRS: immune‑related risk score
lncRNAs: long non-coding RNAs
LGG: low-grade glioma
LASSO: Least Absolute Shrinkage and Selection Operator
PCA: principal component analysis
ROC: Receiver Operating Characteristic
RS: risk scores
TCGA: The Cancer Genome Atlas
TIMER: Tumor Immune Estimation Resource
