# Supplementary material for: Development and Validation of an Immune-Related Long Non-coding RNA Prognostic Model in Glioma
Source: J Cancer. 2021 May 19;12(14):4264–76. doi: 10.7150/jca.53831 (PMC8176429; doi:10.7150/jca.53831)
Supplement: Supplementary file 1 — Supplementary tables. [file jcav12p4264s1.pdf]

Supplementary table 1 Immune related lncRNAs co-expression analysis

Immune Gene	lncRNA	cor	pvalue	Regulation
APOBEC3F	CARD8-AS1	0.705144401	5.11E-106	postive
APOBEC3F	LINC00900	0.718803729	5.46E-112	postive
APOBEC3G	AC147651.3	0.720306208	1.15E-112	postive
APOBEC3G	CARD8-AS1	0.741364336	1.14E-122	postive
APOBEC3G	PSMB8-AS1	0.717875459	1.43E-111	postive
ARHGDIB	AC098613.1	0.797669316	5.33E-155	postive
ARHGDIB	AC138207.5	0.771675118	6.41E-139	postive
ARHGDIB	AC145098.1	0.715605908	1.47E-110	postive
ARHGDIB	CARD8-AS1	0.748075364	4.56E-126	postive
ARHGDIB	LINC01150	0.846964223	3.87E-193	postive
ARHGDIB	PCED1B-AS1	0.909974161	2.75E-268	postive
BLNK	AC090559.1	0.741358613	1.14E-122	postive
BLNK	AC098613.1	0.734313337	3.24E-119	postive
BLNK	LINC01150	0.705879595	2.49E-106	postive
C2	AC145098.1	0.70621531	1.79E-106	postive
CCR1	AC008760.2	0.7187544	5.75E-112	postive
CCR1	AC090559.1	0.71537425	1.86E-110	postive
CCR1	AC098613.1	0.72230464	1.41E-113	postive
CCR1	AC110995.1	0.784028223	2.78E-146	postive
CCR1	AC145098.1	0.759724874	3.17E-132	postive
CCR1	LINC01150	0.744413609	3.35E-124	postive
CCR1	PCED1B-AS1	0.722379622	1.30E-113	postive
CCR5	AC008760.2	0.70157668	1.63E-104	postive
CCR5	AC098613.1	0.717158712	2.99E-111	postive
CCR5	AC145098.1	0.806793733	3.26E-161	postive
CCR5	LINC01150	0.720930531	5.96E-113	postive
CD164	CARD8-AS1	0.710153573	3.62E-108	postive
CD22	AC009063.2	0.721487325	3.33E-113	postive
CD22	AC016597.1	0.888680328	6.95E-238	postive
CD22	AC018647.1	0.773593535	4.94E-140	postive
CD22	AL023284.4	0.770844845	1.93E-138	postive
CD22	HPN-AS1	0.741357995	1.14E-122	postive
CD22	NTM-AS1	0.791749961	3.89E-151	postive
CD24	AL355297.4	0.706904958	9.08E-107	postive
CD24	LINC02526	0.838631108	8.36E-186	postive
CD4	AC002091.1	0.757729501	3.81E-131	postive
CD4	AC090559.1	0.787525554	1.86E-148	postive
CD4	AC110995.1	0.760675551	9.61E-133	postive
CD4	AC138207.5	0.743875963	6.26E-124	postive
CD4	AC145098.1	0.777577478	2.23E-142	postive
CD4	AL133371.2	0.715787423	1.22E-110	postive
CD4	AL161785.1	0.753969387	3.86E-129	postive

CD4	LINC01150	0.790258034	3.50E-150	postive
CD4	PCED1B-AS1	0.779915323	8.88E-144	postive
CD74	AC138207.5	0.709102562	1.03E-107	postive
CD74	AC145098.1	0.725492078	4.81E-115	postive
CD74	LINC01150	0.745477327	9.69E-125	postive
CD74	PCED1B-AS1	0.760234647	1.67E-132	postive
CD86	AC002091.1	0.745398653	1.06E-124	postive
CD86	AC090559.1	0.759425908	4.61E-132	postive
CD86	AC098613.1	0.779000474	3.15E-143	postive
CD86	AC110995.1	0.711663439	7.99E-109	postive
CD86	AC145098.1	0.715157148	2.32E-110	postive
CD86	AL133371.2	0.71978006	1.98E-112	postive
CD86	AL161785.1	0.745794944	6.69E-125	postive
CD86	LINC01150	0.81087443	4.22E-164	postive
CD86	PCED1B-AS1	0.808496507	2.07E-162	postive
CDC42	CARD8-AS1	0.74710146	1.44E-125	postive
CDC42	LINC00339	0.75886268	9.30E-132	postive
CKLF	CARD8-AS1	0.768628529	3.56E-137	postive
CKLF	CYTOR	0.713624251	1.10E-109	postive
CKLF	LINC00339	0.718759636	5.72E-112	postive
CKLF	MIR4435-2HG	0.70067002	3.89E-104	postive
CLEC7A	AC098613.1	0.715407636	1.80E-110	postive
CLEC7A	AC138207.4	0.71669061	4.83E-111	postive
CLEC7A	AC145098.1	0.756581395	1.57E-130	postive
CLEC7A	LINC01150	0.74185993	6.43E-123	postive
CLEC7A	PCED1B-AS1	0.749152299	1.27E-126	postive
CRHR1	AF106564.1	0.787283441	2.64E-148	postive
CTSC	PCED1B-AS1	0.723040429	6.49E-114	postive
CTSS	AC002091.1	0.736168221	4.10E-120	postive
CTSS	AC098613.1	0.755208855	8.50E-130	postive
CTSS	AC138207.4	0.700489724	4.63E-104	postive
CTSS	AC138207.5	0.748916897	1.68E-126	postive
CTSS	AC145098.1	0.756611954	1.52E-130	postive
CTSS	CARD8-AS1	0.713325121	1.49E-109	postive
CTSS	LINC01150	0.792055415	2.47E-151	postive
CTSS	PCED1B-AS1	0.800706648	4.95E-157	postive
DOCK2	AC002091.1	0.767326197	1.95E-136	postive
DOCK2	AC090559.1	0.901453138	2.72E-255	postive
DOCK2	AC093627.7	0.737711068	7.24E-121	postive
DOCK2	AC110995.1	0.727653497	4.73E-116	postive
DOCK2	AF127936.1	0.712175089	4.77E-109	postive
DOCK2	AL133371.2	0.727312226	6.84E-116	postive
DOCK2	AL161785.1	0.793248125	4.21E-152	postive
DOCK2	LINC01150	0.726192951	2.27E-115	postive

EBI3	AC090559.1	0.710280161	3.19E-108	postive
EBI3	AL161785.1	0.749975955	4.76E-127	postive
FCGR1A	AC098613.1	0.814178566	1.72E-166	postive
FCGR1A	AC138207.5	0.770142591	4.87E-138	postive
FCGR1A	AL355922.1	0.731671179	6.00E-118	postive
FCGR1A	LINC01150	0.86369323	2.79E-209	postive
FCGR1A	PCED1B-AS1	0.90456717	6.73E-260	postive
FCGR3A	AC008760.2	0.740036628	5.18E-122	postive
FCGR3A	AC098613.1	0.724727027	1.09E-114	postive
FCGR3A	AC138207.5	0.713585243	1.15E-109	postive
FCGR3A	AC145098.1	0.787986879	9.56E-149	postive
FCGR3A	AL355922.1	0.70568423	3.01E-106	postive
FCGR3A	LINC01150	0.783357333	7.18E-146	postive
FCGR3A	PCED1B-AS1	0.849409663	2.25E-195	postive
FCGRT	AC138207.5	0.730671243	1.79E-117	postive
FCGRT	CARD8-AS1	0.735259795	1.13E-119	postive
FCGRT	PCED1B-AS1	0.781927662	5.37E-145	postive
FYB1	AC008760.2	0.700409957	5.00E-104	postive
FYB1	AC090559.1	0.811068532	3.06E-164	postive
GPR65	AC008760.2	0.708239689	2.43E-107	postive
GPR65	AC098613.1	0.816129043	6.34E-168	postive
GPR65	AC138207.5	0.734795372	1.90E-119	postive
GPR65	AC145098.1	0.785418152	3.84E-147	postive
GPR65	CARD8-AS1	0.782969566	1.24E-145	postive
GPR65	LINC01150	0.823067846	3.64E-173	postive
GPR65	PCED1B-AS1	0.846696156	6.77E-193	postive
HAMP	AC008760.2	0.720906123	6.12E-113	postive
HAMP	AC098613.1	0.734837789	1.81E-119	postive
HAMP	AL355922.1	0.796337035	4.05E-154	postive
HAMP	LINC01150	0.729039368	1.06E-116	postive
HAMP	PCED1B-AS1	0.803559672	5.66E-159	postive
HCLS1	AC002091.1	0.733956578	4.82E-119	postive
HCLS1	AC090559.1	0.733472884	8.24E-119	postive
HCLS1	AC145098.1	0.738133394	4.49E-121	postive
HCLS1	AL133371.2	0.734470068	2.73E-119	postive
HCLS1	AL161785.1	0.7445107	2.99E-124	postive
HCLS1	LINC01150	0.71868419	6.18E-112	postive
HCLS1	PCED1B-AS1	0.736705561	2.24E-120	postive
HDAC4	AC009227.1	0.73071245	1.71E-117	postive
HDAC4	PAXBP1-AS1	0.717072138	3.27E-111	postive
HDAC4	SLC6A1-AS1	0.707659082	4.32E-107	postive
HELLS	AC012073.1	0.711928335	6.12E-109	postive
HELLS	TMPO-AS1	0.711711435	7.61E-109	postive
IGSF6	AC098613.1	0.726627353	1.43E-115	postive

IGSF6	AC138207.5	0.722925305	7.33E-114	postive
IGSF6	AL161785.1	0.713978704	7.70E-110	postive
IGSF6	LINC01150	0.752924084	1.37E-128	postive
IGSF6	PCED1B-AS1	0.715927523	1.06E-110	postive
IL10RB	AC073611.1	0.724035253	2.27E-114	postive
IL10RB	AL445524.1	0.724753357	1.06E-114	postive
IL10RB	CARD8-AS1	0.717561845	1.97E-111	postive
IL16	AC002091.1	0.733017712	1.36E-118	postive
IL16	AC090559.1	0.811987428	6.69E-165	postive
IL16	AC098613.1	0.727871767	3.74E-116	postive
IL16	AC145098.1	0.702228822	8.69E-105	postive
IL16	AL133371.2	0.708796391	1.40E-107	postive
IL16	AL161785.1	0.786519228	7.94E-148	postive
IL16	LINC01150	0.774291661	1.93E-140	postive
IL16	PCED1B-AS1	0.759345436	5.09E-132	postive
IL18	AC098613.1	0.759927404	2.46E-132	postive
IL18	AC138207.5	0.71291931	2.25E-109	postive
IL18	AL161785.1	0.755227444	8.31E-130	postive
IL18	LINC01150	0.7842163	2.13E-146	postive
IL18	PCED1B-AS1	0.801115095	2.62E-157	postive
IL4R	AC145098.1	0.737783127	6.67E-121	postive
IL6R	AC090559.1	0.733344085	9.50E-119	postive
IRF8	AC090559.1	0.749338461	1.02E-126	postive
IRF8	AC110995.1	0.715196133	2.23E-110	postive
IRF8	AF127936.1	0.703077519	3.82E-105	postive
IRF8	AL161785.1	0.768525145	4.08E-137	postive
IRF8	LINC01150	0.703702909	2.08E-105	postive
ITGB2	AC002091.1	0.756175058	2.60E-130	postive
ITGB2	AC008760.2	0.737180459	1.32E-120	postive
ITGB2	AC090559.1	0.768091176	7.19E-137	postive
ITGB2	AC098613.1	0.723082391	6.21E-114	postive
ITGB2	AC138207.5	0.764942849	4.24E-135	postive
ITGB2	AC145098.1	0.813063514	1.12E-165	postive
ITGB2	AL133371.2	0.722935153	7.26E-114	postive
ITGB2	AL161785.1	0.785882456	1.98E-147	postive
ITGB2	LINC01150	0.807435703	1.16E-161	postive
ITGB2	PCED1B-AS1	0.80263106	2.44E-158	postive
KAT6A	AC010834.3	0.729039217	1.06E-116	postive
KAT6A	AC068152.1	0.735078684	1.38E-119	postive
KAT6A	AC108449.2	0.817672177	4.53E-169	postive
KMT2A	AC009227.1	0.707164715	7.03E-107	postive
KMT2A	AC010834.3	0.762832937	6.28E-134	postive
KMT2A	AC108449.2	0.770687967	2.37E-138	postive
KMT2A	AL157392.3	0.730086527	3.39E-117	postive

KMT2A	ZBTB20-AS4	0.749050566	1.43E-126	postive
LAT2	AC090559.1	0.707308662	6.10E-107	postive
LAT2	AC098613.1	0.800833287	4.06E-157	postive
LAT2	AC138207.5	0.713077868	1.92E-109	postive
LAT2	AL161785.1	0.742904012	1.93E-123	postive
LAT2	LINC01150	0.831666241	5.47E-180	postive
LAT2	PCED1B-AS1	0.829790515	1.82E-178	postive
LCP2	AC145098.1	0.785496168	3.44E-147	postive
LCP2	LINC01150	0.712313772	4.15E-109	postive
LCP2	PCED1B-AS1	0.729772257	4.77E-117	postive
LIG1	AC012073.1	0.725007424	8.06E-115	postive
LIG1	ZIM2-AS1	0.700152363	6.39E-104	postive
LILRB2	AC138207.4	0.731824182	5.07E-118	postive
LILRB2	AC138207.5	0.710092546	3.85E-108	postive
LILRB2	AC145098.1	0.746707764	2.29E-125	postive
LILRB2	PCED1B-AS1	0.729882301	4.24E-117	postive
LRMP	AC090559.1	0.71578741	1.22E-110	postive
LST1	AC098613.1	0.795768965	9.57E-154	postive
LST1	AC138207.5	0.737791875	6.61E-121	postive
LST1	LINC01150	0.781848886	5.99E-145	postive
LST1	PCED1B-AS1	0.873431015	1.06E-219	postive
LY86	AC018755.4	0.700675424	3.87E-104	postive
LY86	AC098613.1	0.836269506	8.41E-184	postive
LY86	AC110995.1	0.708695611	1.55E-107	postive
LY86	AC138207.5	0.791355476	6.96E-151	postive
LY86	AL161785.1	0.752402091	2.58E-128	postive
LY86	LINC01150	0.860596462	3.91E-206	postive
LY86	PCED1B-AS1	0.883227	4.32E-231	postive
LYN	AC008760.2	0.700123093	6.58E-104	postive
LYN	AC090559.1	0.724495268	1.39E-114	postive
LYN	AC098613.1	0.783784449	3.92E-146	postive
LYN	AC145098.1	0.758488013	1.48E-131	postive
LYN	LINC01150	0.806546288	4.85E-161	postive
LYN	PCED1B-AS1	0.785575315	3.07E-147	postive
MAL	AC009063.2	0.801606509	1.22E-157	postive
MAL	AC016597.1	0.860816604	2.35E-206	postive
MAL	AC018647.1	0.759332963	5.17E-132	postive
MAL	AL023284.4	0.782931419	1.31E-145	postive
MAL	AL138881.1	0.706610253	1.21E-106	postive
MAL	AL358216.1	0.701473353	1.80E-104	postive
MAL	HPN-AS1	0.81367092	4.03E-166	postive
MAL	NTM-AS1	0.805372315	3.18E-160	postive
MBP	AC009063.2	0.847215342	2.29E-193	postive
MBP	AC016597.1	0.882463691	3.63E-230	postive

MBP	AC018647.1	0.802249256	4.45E-158	postive
MBP	AL023284.4	0.766623553	4.85E-136	postive
MBP	AL138881.1	0.725727371	3.74E-115	postive
MBP	AL358216.1	0.706991948	8.34E-107	postive
MBP	HPN-AS1	0.779796342	1.05E-143	postive
MBP	LINC00320	0.732754697	1.82E-118	postive
MBP	NTM-AS1	0.783234334	8.54E-146	postive
MLF1	ERICH6-AS1	0.782772676	1.64E-145	postive
MLF1	NRAV	0.760352272	1.44E-132	postive
NCF4	AC098613.1	0.736153066	4.17E-120	postive
NCF4	AC110995.1	0.750644166	2.14E-127	postive
NCF4	AC138207.4	0.705921495	2.39E-106	postive
NCF4	AC138207.5	0.838305709	1.58E-185	postive
NCF4	AC145098.1	0.722023605	1.90E-113	postive
NCF4	AL161785.1	0.743958746	5.69E-124	postive
NCF4	LINC01150	0.810030347	1.69E-163	postive
NCF4	PCED1B-AS1	0.882063393	1.10E-229	postive
NCOA6	AC005288.1	0.742073983	5.02E-123	postive
NFAM1	AC145098.1	0.794483341	6.63E-153	postive
POU2F2	AC090559.1	0.715961392	1.02E-110	postive
PRELID1	AL445524.1	0.71602412	9.57E-111	postive
PRELID1	MIR4435-2HG	0.70276055	5.19E-105	postive
PSMB10	PCED1B-AS1	0.71614032	8.50E-111	postive
PSMB10	PSMB8-AS1	0.704799669	7.16E-106	postive
PTAFR	AC002091.1	0.708947287	1.20E-107	postive
PTAFR	AC090559.1	0.799515064	3.13E-156	postive
PTAFR	AC098613.1	0.748631065	2.36E-126	postive
PTAFR	AC110995.1	0.742805646	2.16E-123	postive
PTAFR	AL161785.1	0.792251077	1.85E-151	postive
PTAFR	LINC01150	0.774954122	7.91E-141	postive
PTPRC	AC090559.1	0.823193107	2.91E-173	postive
PTPRC	AC145098.1	0.744487559	3.08E-124	postive
PTPRC	LINC01150	0.713092871	1.89E-109	postive
RASGRP4	AC002091.1	0.774984606	7.59E-141	postive
RASGRP4	AC090559.1	0.821304026	8.22E-172	postive
RASGRP4	AC110995.1	0.756871475	1.10E-130	postive
RASGRP4	AC138207.5	0.738924034	1.84E-121	postive
RASGRP4	AC145098.1	0.72820771	2.60E-116	postive
RASGRP4	AL133371.2	0.760550138	1.13E-132	postive
RASGRP4	AL161785.1	0.848240435	2.67E-194	postive
RASGRP4	LINC01150	0.789489908	1.08E-149	postive
RASGRP4	PCED1B-AS1	0.73753323	8.85E-121	postive
SART1	AC010536.3	0.71279331	2.56E-109	postive
SART1	AP001505.1	0.705378682	4.06E-106	postive

SCIN	AC008760.2	0.744285837	3.89E-124	postive
SCIN	AC090559.1	0.771495623	8.14E-139	postive
SCIN	LINC01150	0.737517544	9.00E-121	postive
SCIN	PCED1B-AS1	0.714843507	3.20E-110	postive
SEMA4D	AC016597.1	0.797304085	9.31E-155	postive
SEMA4D	AC018647.1	0.839590017	1.26E-186	postive
SEMA4D	NTM-AS1	0.71634534	6.89E-111	postive
SPI1	AC002091.1	0.723382275	4.52E-114	postive
SPI1	AC008760.2	0.714509428	4.49E-110	postive
SPI1	AC090559.1	0.702891985	4.57E-105	postive
SPI1	AC098613.1	0.744191731	4.34E-124	postive
SPI1	AC110995.1	0.720040526	1.51E-112	postive
SPI1	AC138207.5	0.809394003	4.80E-163	postive
SPI1	AC145098.1	0.773264543	7.69E-140	postive
SPI1	AL161785.1	0.779886976	9.23E-144	postive
SPI1	LINC01150	0.827658214	9.24E-177	postive
SPI1	PCED1B-AS1	0.840984904	7.82E-188	postive
ST6GAL1	AC090559.1	0.736767773	2.09E-120	postive
ST6GAL1	AC093627.7	0.735389525	9.79E-120	postive
SYK	AC002091.1	0.778652083	5.09E-143	postive
SYK	AC090559.1	0.884962739	3.24E-233	postive
SYK	AC093627.7	0.726065816	2.60E-115	postive
SYK	AC098613.1	0.7296192	5.64E-117	postive
SYK	AC110995.1	0.767861981	9.69E-137	postive
SYK	AC138207.5	0.714772661	3.44E-110	postive
SYK	AC145098.1	0.708513883	1.85E-107	postive
SYK	AL133371.2	0.766052587	1.01E-135	postive
SYK	AL161785.1	0.842291639	5.66E-189	postive
SYK	LINC01150	0.809938868	1.97E-163	postive
SYK	PCED1B-AS1	0.77059968	2.67E-138	postive
TAZ	AC004148.2	0.763191334	3.98E-134	postive
TAZ	AC005785.1	0.783282204	7.98E-146	postive
TAZ	AC009065.8	0.769062228	2.02E-137	postive
TAZ	AC009283.1	0.735520523	8.46E-120	postive
TAZ	AC016773.1	0.757673338	4.08E-131	postive
TAZ	AC069281.2	0.768481309	4.32E-137	postive
TAZ	AC092119.2	0.717915589	1.37E-111	postive
TAZ	AC092171.4	0.726238319	2.17E-115	postive
TAZ	AC126118.1	0.727369081	6.43E-116	postive
TAZ	AL022328.2	0.705335309	4.24E-106	postive
TAZ	AL109811.3	0.74287881	1.99E-123	postive
TAZ	AL117379.1	0.728861938	1.28E-116	postive
TAZ	ARHGAP27P1-BPTFP1-KPNA2P3	0.716357414	6.80E-111	postive
TAZ	ASMTL-AS1	0.748106922	4.39E-126	postive

TAZ	MHENCR	0.702020554	1.06E-104	postive
TAZ	SLC25A25-AS1	0.712608039	3.08E-109	postive
TCF12	AC009227.1	0.72668548	1.34E-115	postive
TGFB2	TGFB2-AS1	0.947344465	0	postive
TLR7	AC090559.1	0.85923014	9.03E-205	postive
TLR7	AC110995.1	0.751822815	5.20E-128	postive
TLR7	AF127936.1	0.705512389	3.57E-106	postive
TLR7	AL161785.1	0.75886111	9.32E-132	postive
TLR7	LINC01150	0.732161769	3.50E-118	postive
TLR8	AC145098.1	0.744647676	2.55E-124	postive
TREM2	AC098613.1	0.718134712	1.09E-111	postive
TREM2	AC138207.5	0.706042588	2.12E-106	postive
TREM2	AL161785.1	0.713572553	1.16E-109	postive
TREM2	LINC01150	0.778833097	3.97E-143	postive
TREM2	PCED1B-AS1	0.786317308	1.06E-147	postive
VIPR1	AC004816.1	0.759169346	6.35E-132	postive
VIPR1	AC008780.2	0.820797545	2.00E-171	postive
VIPR1	AC015468.3	0.753001311	1.25E-128	postive
VIPR1	AC015967.1	0.770097546	5.17E-138	postive
VIPR1	AC061961.1	0.783443168	6.36E-146	postive
VIPR1	AC104072.1	0.802831048	1.79E-158	postive
VIPR1	AC110491.1	0.737259845	1.20E-120	postive
VIPR1	AC125616.1	0.791732665	3.99E-151	postive
VIPR1	AC138649.1	0.765946759	1.16E-135	postive
VIPR1	AL021395.1	0.855121839	9.35E-201	postive
VIPR1	AL356479.1	0.700922517	3.06E-104	postive
VIPR1	AP003355.2	0.874750995	3.53E-221	postive
VIPR1	ATP2B1-AS1	0.745021282	1.65E-124	postive
VIPR1	DLGAP1-AS4	0.859761324	2.67E-205	postive
VIPR1	LINC00507	0.795138677	2.48E-153	postive
VIPR1	MIR600HG	0.764802499	5.07E-135	postive
VIPR1	PART1	0.862762667	2.51E-208	postive
VIPR1	RFPL1S	0.81671186	2.35E-168	postive
VIPR1	SLC26A4-AS1	0.853065327	8.60E-199	postive
VTN	AP006623.1	0.716105036	8.81E-111	postive
VTN	ZNF213-AS1	0.711646395	8.12E-109	postive
WAS	AC090559.1	0.729047537	1.05E-116	postive
WAS	AC098613.1	0.723411618	4.39E-114	postive
WAS	AC138207.5	0.759877349	2.62E-132	postive
WAS	AC145098.1	0.722609047	1.02E-113	postive
WAS	AL161785.1	0.77868767	4.85E-143	postive
WAS	LINC01150	0.79015628	4.06E-150	postive
WAS	PCED1B-AS1	0.791766991	3.79E-151	postive
ZNF675	AC011477.3	0.770324863	3.83E-138	postive

Supplementary table 2 Univariate COX analysis identified immune related lncRNAs

ID	Hazard ratio (HR)	HR.95L	HR.95H	pvalue
CYTOR	2.272647	2.04999	2.519487	6.74E-55
TGFB2-AS1	1.586601	1.43489	1.754351	2.22E-19
TMPO-AS1	2.182833	1.612303	2.95525	4.41E-07
AC090559.1	1.481147	1.232905	1.779372	2.71E-05
AC008760.2	1.913823	1.679449	2.180904	2.06E-22
AC018647.1	0.525794	0.432296	0.639514	1.24E-10
LINC01150	2.108918	1.729539	2.571515	1.65E-13
ERICH6-AS1	1.963907	1.638012	2.354642	3.09E-13
AL021395.1	0.44512	0.319661	0.619818	1.65E-06
AC138207.4	3.608729	2.729836	4.770588	2.03E-19
AL022328.2	0.503879	0.395868	0.641361	2.57E-08
PAXBP1-AS1	0.150753	0.104745	0.216969	2.31E-24
AC145098.1	3.471031	2.790766	4.317113	5.02E-29
AL161785.1	1.429925	1.183244	1.728034	0.000214
AL355297.4	0.455504	0.329286	0.630102	2.04E-06
AC009227.1	0.149051	0.108052	0.205608	4.24E-31
AC108449.2	0.586626	0.490135	0.702112	5.98E-09
AL023284.4	0.677317	0.577876	0.79387	1.51E-06
AP006623.1	0.315043	0.21047	0.471573	2.00E-08
CARD8-AS1	3.414321	2.803478	4.158258	2.75E-34
PSMB8-AS1	2.50268	2.096438	2.987641	3.27E-24
AC018755.4	1.476234	1.239305	1.758458	1.28E-05
NRAV	3.392108	2.798168	4.112117	1.64E-35
PART1	0.628179	0.477248	0.826844	0.000913
ZIM2-AS1	2.040535	1.63514	2.546436	2.77E-10
LINC00339	3.511252	2.793495	4.413428	5.05E-27
AC004148.2	0.681164	0.557478	0.832293	0.000173
AC010536.3	0.12729	0.085712	0.189039	1.69E-24
PCED1B-AS1	2.007918	1.707411	2.361313	3.53E-17
AP001505.1	0.479898	0.391684	0.587979	1.40E-12
AC010834.3	0.307982	0.225677	0.420305	1.14E-13
AC012073.1	3.289475	2.602098	4.158432	2.37E-23
AC015967.1	0.130933	0.085279	0.20103	1.49E-20
AC138649.1	0.637297	0.508291	0.799045	9.46E-05
AC073611.1	3.473073	2.799318	4.30899	1.11E-29
AC126118.1	0.409833	0.292857	0.573533	1.97E-07
LINC00900	5.890004	4.471243	7.75895	1.83E-36
AC110491.1	0.449834	0.333405	0.606921	1.72E-07
AC092171.4	0.650166	0.507309	0.83325	0.000671
AL445524.1	4.578718	3.66869	5.714481	2.79E-41
ZBTB20-AS4	0.143213	0.098213	0.208833	5.63E-24
AC068152.1	0.45629	0.310589	0.670342	6.39E-05

AL355922.1	1.83687	1.594133	2.116568	4.15E-17
ZNF213-AS1	0.399205	0.296915	0.536735	1.20E-09
AC011477.3	0.474073	0.369369	0.608457	4.58E-09
AC092119.2	0.548599	0.385707	0.780283	0.000837
SLC6A1-AS1	0.258098	0.188915	0.352617	1.78E-17
AF106564.1	0.269595	0.195144	0.37245	1.87E-15
AP003355.2	0.648685	0.510361	0.824501	0.000405
AC138207.5	1.712105	1.435159	2.042492	2.33E-09
AC061961.1	0.46065	0.345685	0.613849	1.21E-07
LINC00320	0.552108	0.476939	0.639125	1.79E-15
MIR600HG	0.362379	0.279533	0.469779	1.79E-14
RFPL1S	0.568468	0.473713	0.682177	1.27E-09
MIR4435-2HG	2.344239	2.081627	2.639981	7.24E-45
SLC25A25-AS1	0.571207	0.427503	0.763216	0.000152
AC098613.1	2.063407	1.724142	2.469431	2.71E-15
AC125616.1	0.272939	0.181533	0.41037	4.36E-10
AC009283.1	0.493242	0.385917	0.630415	1.65E-08
AL133371.2	1.813064	1.442225	2.279257	3.46E-07
AC002091.1	2.546407	1.878572	3.451658	1.71E-09
AL157392.3	0.088843	0.062626	0.126035	6.14E-42
AC147651.3	2.769052	2.391742	3.205885	2.74E-42
